# A research protocol using ECG monitoring for detection of palpitations-associated arrhythmias in breast cancer survivors

**DOI:** 10.1371/journal.pone.0338932

**Published:** 2025-12-26

**Authors:** Ying Sheng, Matthew R. Fleming, Jessica Mezzanotte-Sharpe, Nethmi S. Hewavithana, Sadie B. Sommer, Mary S. Dietrich, Janet S. Carpenter

**Affiliations:** 1 School of Nursing, Vanderbilt University, Nashville, Tennessee, United States of America; 2 Vanderbilt University Medical Center, Nashville, Tennessee, United States of America; 3 School of Nursing, Indiana University, Indianapolis, Indiana, United States of America; Oswaldo Cruz Foundation, BRAZIL

## Abstract

Palpitations are common but understudied in breast cancer survivors (BCS). Palpitations may relate to severe arrhythmias, resulting in life-threatening events or cardiac death. However, how palpitations relate to arrhythmias and electrocardiogram abnormalities is unknown. The purpose of the proposed project is to demonstrate the feasibility of using wearable electrocardiogram (ECG) monitors in BCS to detect and characterize arrhythmias associated with self-reported palpitations and to gain a comprehensive understanding of palpitations using an investigator-designed Palpitations Assessment Tool (PAT). We will conduct a prospective cohort study of 84 BCS (54 BCS with palpitations and 30 without). Eligible participants will include breast cancer patients who completed chemotherapy at least six months ago and no more than three years ago. Palpitations and arrhythmias will be recorded using an ECG monitor for two 7-day periods, one month apart. Feasibility, acceptability, and retention, as measured by completion of the PAT and monitored wearing for 7 days, will be evaluated using frequency distribution in recruitment and retention logs. We will generate descriptive summaries of the prevalence of primary outcomes, including frequency of palpitations and changes in cardiac rate and rhythm, and examine associations between these outcomes.

## Introduction

Over the last decade, data from various studies have revealed that 15% to 48% of breast cancer survivors (BCS) experience palpitations, the cardiac sensation of heart “pounding or racing” that many patients find distressing [1–3]. Palpitations are clinically important, as they are linked to increased healthcare utilization and, in some instances, potentially life-threatening arrhythmias. In the general population, palpitations account for 16% of primary care provider visits [4] and are the second leading reason for cardiologist visits [4]. Frequent or disruptive palpitations, particularly those that interfere with sleep or occur during work, are associated with an increased risk of cardiac arrhythmias [5,6].

In both women with and without breast cancer, palpitations are also associated with a higher symptom burden, including more severe menopausal symptoms [3], increased anxiety [7], fatigue [7], poor sleep [7,8], depressive symptoms [7–10], and stress [8], all of which negatively impact quality of life [3,7,8,10]. Despite their prevalence and clinical significance, palpitations remain understudied and under-treated in this population [11].

To date, two published studies [1,2], several secondary analyses, and a pilot survey from our team [3,7,12–14] have investigated palpitations in breast cancer patients. Additional research conducted among German [2] and Malaysian [1] BCS receiving oral endocrine therapy reported variable prevalence rates ranging from 17.7% to 48%, depending on treatment duration, but did not examine associated factors. Work by our team focused on women prior to breast cancer surgery [3,7,12,13], and found that 15.1% had palpitations in the past week. Those with palpitations had lower household income and functional status [3], higher comorbidity and symptom burden, poorer quality of life [7], and variations in cytokine [12] and neurotransmitter [13] genes. However, the dataset lacked cardiovascular risk factors (e.g., alcohol intake, smoking) and information on palpitations post treatment, limiting its generalizability.

Importantly, none of these studies have investigated electrocardiogram (ECG) recordings in patients with palpitations and their relationship with the occurrence of arrhythmias. Research on ECG recordings in breast cancer patients with palpitations is limited. With the emergence of cardio-oncology, several reviews [11,15–17] have found that both cardiac arrhythmias (e.g., atrial fibrillation, sinus tachycardia, supraventricular tachycardia) and cancer-specific causes (e.g., chemotherapy, cardiac myomas) are common causes of palpitations in cancer patients. Given the increasing overlap between cancer and cardiovascular disease [11], ECG monitoring with demonstration of symptom-rhythm correlation is recommended for patients with recurrent palpitations to detect cardiac arrhythmias, as clinical examinations alone often fail to identify clinically significant arrhythmias [6]. One study reported arrhythmias in 65% of adult and mostly female patients with palpitations or dizziness, with 29% being clinically relevant [18]. Another study found arrhythmias in 81% of patients using a 12-lead ECG, with atrial fibrillation being the most common (77%) [5]. However, neither study included breast cancer patients, despite growing recognition of cardiac effects of medications [11,15–17]. In spite of the increasing recognition of the co-occurrence of cancer and cardiovascular disease [11,19], palpitations are often attributed to psychosomatic origins (e.g., anxiety) rather than cardiac causes [20].

In addition, there is limited evidence describing other non-rhythmic mechanisms underlying palpitations in women. Biomarkers and gene variations may contribute to the development of palpitations. Serum cardiac biomarkers, such as a new rise in Troponin I/T, B-type natriuretic peptide (BNP), or N-terminal B-type natriuretic peptide (NT-proBNP) have been well documented as indicators of cancer therapy-related cardiac dysfunction [19,21]. Palpitations, along with dyspnea, chest pain, peripheral edema, and fatigue, are the five most common chemotherapy-related cardiotoxicity symptoms [22]. These biomarkers may also be associated with the occurrence of palpitations, highlighting the potential link between biological markers and this symptom. Our secondary analyses have revealed that palpitations are associated with cytokine gene polymorphisms, neurotransmitter genes, and potassium channel genes in breast cancer patients before surgery [12,13]. No other genetic studies have examined palpitations in this population, and only two studies in women without cancer have reported null findings [23,24]. Further investigation of genotypic factors may help elucidate the molecular mechanisms underlying the development of palpitations.

Palpitations are also common in conditions such as COVID-19 infection, likely due to sympathetic activation and pro-inflammatory cytokine release [25]. Chronic inflammation, including cytokine deregulation, may contribute to symptom development [26,27]. Neurotransmitters such as epinephrine, norepinephrine, serotonin, and gamma-aminobutyric acid, as well as potassium channels, play key roles in both neural and cardiovascular functions, including cardiac repolarization and cellular excitability [28]. Genetic variations in these pathways have been linked to cardiovascular diseases [29–34] and may represent an additional, non-rhythmic mechanism contributing to palpitations.

To address these gaps, our study will include a comparison group of BCS without palpitations, providing valuable insight into the underlying mechanisms of palpitations in BCS. This will enable us to identify differences in symptoms, ECG findings, and biological markers, such as inflammatory markers, potassium genes, cardiovascular markers, cortisol, and thyroid hormones between those with and without palpitations.

## Materials and methods

### Aims

The primary aim of this feasibility study is to evaluate the use of a novel wearable ECG monitor to detect and characterize ECG abnormalities associated with the sensation of palpitations in BCS. The secondary aim is to gain a comprehensive understanding of the palpitations experience using an investigator-designed Palpitations Assessment Tool (PAT). We will also bank blood samples for future studies to explore biological differences between BCS with and without palpitations.

### Study design

We will conduct a prospective cohort study using 7-day continuous ECG monitoring to detect palpitations and their associated rate/rhythm changes (arrhythmias). We will use our designed PAT to measure the frequency, distress, and associated cardiopulmonary symptoms of self-reported palpitations (e.g., chest pain, dizziness, or unexplained shortness of breath). Additionally, a group of breast cancer patients without palpitations will be included to serve as a comparison group to examine the underlying mechanisms of palpitations.

### Study setting

The study will be conducted in a metropolitan city in the mid-south region that is home to a comprehensive cancer center.

### Sample size

We aim to enroll 100 participants, including 60 with self-reported palpitations and 40 without, to provide adequate sample sizes for our primary comparative analyses.

### Inclusion criteria

Inclusion criteria for all participants will be: (1) women aged ≥18 years, (2) willing to complete all study procedures, (3) peri- or postmenopausal per Stages of Reproductive Aging Workshop +10 (STRAW+10) criteria [35,36], (4) diagnosis of breast cancer, and (5) for those who received intravenous chemotherapy for breast cancer, the treatment must have been completed at least six months ago and no more than three years ago. For the palpitations group, women must have a history of self-reported palpitations at least once during the 2 weeks preceding study enrollment and at least once during the 2-week diary period. For the non-palpitations group (comparison), women must report no episodes of palpitations within the past 6 months and none during the 2-week diary period.

### Exclusion criteria

Exclusion criteria will be (1) premenopausal**,** pregnant**,** or breastfeeding; (2) having a diagnosis of a major psychiatric disorder (e.g., schizophrenia, psychosis); or (3) medical history of arrhythmias (with the exception of sinus bradycardia, sinus arrhythmia or sinus tachycardia)**,** heart attack or stroke or congenital heart disease**,** heart failure**,** or permanent pacemaker. Participants will also be excluded if they are (4) taking antiarrhythmic drugs (with the exceptions of ß- blockers, diltiazem, and verapamil); (5) have known serious skin allergies or sensitivities to adhesive materials (ECG monitors are contraindicated); (6) have visual evidence of skin conditions or wounds that could interfere with adherence between the skin and ECG monitor; or (7) have undergone recent surgery or radiation therapy that precludes ability to wear ECG monitor.

### Recruitment strategies

We aim to recruit a total of 130 participants (80 BCS with palpitations and 50 without) from a metropolitan city in the mid-South region using multiple recruitment approaches. Study brochures will be posted in clinics, and physician collaborators will introduce the study to eligible patients during clinical visits. A study-specific reporting workbench report will be used to identify potential participants with active patient portal accounts. Individuals who select the “I’m Interested” option will receive an email with a REDCap (Research Electronic Data Capture) [37,38] prescreening e-consent link and screening questions. Additional recruitment efforts will utilize ResearchMatch, the Research Notifications Distribution List, and online platforms (e.g., Facebook, LinkedIn) to target potential participants within our site’s catchment area. ResearchMatch is a national, nonprofit, National Institutes of Health (NIH) funded listserv designed to connect eligible participants with Institutional Review Board-approved research studies in the U.S. The Research Notifications Distribution List is an institutional email listserv that allows IRB-approved studies to be shared with individuals who have opted in to receiving research study invitations. All brochures and advertisements will also include a link and QR code to access the same consent and screening questionnaires. Potential participants may complete the screening online via the link, QR code, or by calling the study phone number. Calls will be answered by trained staff or the Principal Investigator (PI), who will address any questions and provide the screening link as needed. With expected attrition (up to 20% from our preliminary work), we expect to have an analysis sample of 100 participants.

### Screening

All prospective participants must complete the prescreening e-consent before proceeding to the screening questionnaire. After completing the questionnaire, individuals will be asked to provide contact information for a follow-up call. Research staff will verify eligibility for either the palpitations or non-palpitations groups. Eligible participants will be contacted to schedule a 30-minute call to further review the study. Prior to the call, participants will receive the main consent form and a separate genetic consent form for optional participation in future genomic analyses using the collected blood samples. They will be instructed not to sign until after the call. During the follow-up call, study personnel will review the documents, address questions, and obtain informed consent via the provided REDCap link. Those who consent will also indicate their preferred time to receive the daily symptom diary link for 14 consecutive days via REDCap. Diary completion data will be used to confirm eligibility prior to scheduling their first visit to the research site lab.

### Participant timeline

The duration of participation in this study will be approximately two months. Participants will complete a 14-day diary, which will be used to determine their eligibility as cases, comparisons, or neither for the study. Eligible participants will then wear an ECG monitor for 7 days. One month later, they will wear an ECG monitor for an additional 7 days to capture data, including any cardiac rate/rhythm changes that may not have been recorded during the initial monitoring period. During each 7-day wear period, they will complete symptom questionnaires.

### Sociodemographic and clinical measurements

Demographic data will be collected through a self-reported survey. These will include age, race, ethnicity, marital status, living situation, employment status, education level, and income. Clinical characteristics will include height and weight (for calculation of body mass index), thyroid disease history, history of cardiovascular disease, medications, breast cancer information (e.g., cancer stage, estrogen/progesterone/HER-2 neu receptor status, time of having breast cancer, treatment), use of tamoxifen or aromatase inhibitors, use of hormone therapy, age of menarche, menopausal status, age and reason for periods stopping, pregnancy and child birth information, smoking history, alcohol consumption, and any positive COVID-19 test(s) and associated symptoms. Biometric information, including weight, height, blood pressure, and heart rate will be assessed during the visit by the research staff. For participants who will set up the second ECG monitors at home, a blood pressure monitor will be provided. These participants will self-assess and record their biometric information using a secure REDCap link. The following instruments will also be used to assess additional clinical characteristics.

Functional status will be assessed using the Karnofsky Performance Status (KPS), a valid and reliable 8-item scale modified for use with cancer patients [39]. Participants will rate their functional status on a single item, scored from 30 (I feel severely disabled and need to be hospitalized) to 100 (I feel normal; I have no complaints or symptoms) [39], with higher scores indicating better functional status.

Comorbidity will be evaluated using the Charlson Comorbidity Index (CCI), which classifies the severity of 19 comorbid conditions [40] with each assigned a weight ranging from 1 to 6 based on its severity. The maximum total score is 37 points, with higher scores indicating a greater comorbidity burden.

Physical activity will be measured by the International Physical Activity Questionnaire short form [IPAQ-SF], which captures self-reported physical activity levels, including vigorous, moderate, and walking activity over the past seven days. Menopausal status will be determined using standardized definitions from the Stages of Reproductive Aging Workshop +10 (STRAW+10) criteria [35,36].

### Assessment tools

#### ECG monitoring.

**Fig 1:**
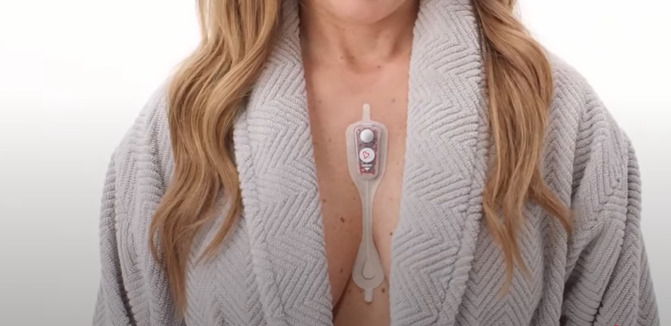
Continuous electrocardiogram monitoring using the Carnation Ambulatory Monitor. The continuous electrocardiogram monitor (Carnation Ambulatory Monitor [CAM Patch], Bardy Diagnostics, part of Baxter) is an FDA-approved, single-use, noninvasive, water-resistant, ambulatory ECG monitoring adhesive patch. Image provided by Baxter International Inc. Carnation Ambulatory Monitor and Bardy Diagnostics are trademarks of Baxter International Inc. or its subsidiaries.

**Fig 2:**
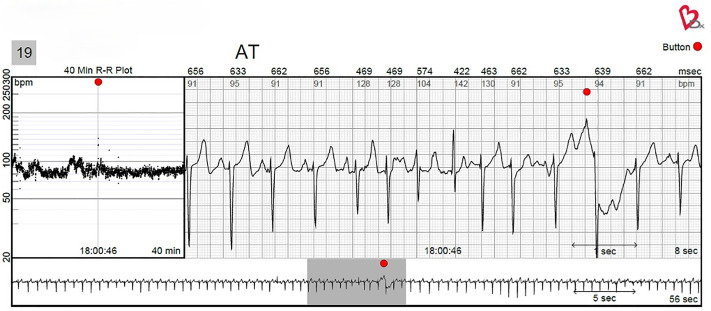
Sample ECG Rhythm Strip. This is a sample ECG strip generated by Bardy Diagnostics from the multi-day CAM patch. Results from a female breast cancer patient with a history of palpitations show that a button press (indicated by the red dot in the top right corner of the strip) co-occurred with an episode of atrial tachycardia (AT noted at the top of the strip). The strip includes a detailed 40-minute R to R plot (left box), an 8-second ECG strip (largest box), and a 56-second ECG strip (bottom box), noting where the 8-second strip occurred (grey shadow). Participant identification number, strip date, and time have been removed to maintain confidentiality. Image provided by Baxter International Inc. Carnation Ambulatory Monitor and Bardy Diagnostics are trademarks of Baxter International Inc. or its subsidiaries. AT = atrial tachycardia, CAM = Carnation Ambulatory Monitor, ECG = electrocardiogram.

We will assess self-reported palpitations and arrhythmias using the Carnation Ambulatory Monitor ([CAM Patch], Bardy Diagnostics, part of Baxter), is an FDA-approved, single-use, noninvasive, water-resistant, ambulatory ECG monitoring adhesive patch (Fig 1). Clinical studies have shown that the CAM patch outperforms the Holter monitor [41] and is more accurate in detecting arrhythmias than the Zio patch [42]. The CAM patch also offers continuous heart monitoring, complemented by expert review from arrhythmia-trained professionals, ensuring high diagnostic accuracy throughout the entire recording period. The CAM patch records heart rhythm and tracks the frequency of palpitations and arrhythmias. The device detects and classifies arrhythmias, such as atrial fibrillation (AF), atrial flutter (AFL), atrial tachycardia (AT), and ventricular tachycardia (VT) [43]. The report identifies arrhythmias, specifies the time and number of events for each specific arrhythmia, and provides an overall arrhythmia burden. Additionally, the report identifies the heart rhythm occurring within 30 seconds prior to and after the time that participants press the event button, confirming whether self-reported palpitations correlate with an arrhythmia. Fig 2 illustrates the correlation between an AT episode and self-reported palpitations, with the red dot representing a single button press made by the participant when they experienced palpitations.

#### Palpitations assessment tool.

The PAT is an investigator-designed tool [14] based on a comprehensive literature review [44]. This 27-item tool evaluates palpitations by first assessing the occurrence of palpitations in the past two weeks using a yes/maybe/no format. If endorsed, participants then respond to 17 predefined descriptors of palpitations sensations, using yes/no responses, along with an open-ended “other” option. Palpitations frequency is assessed with options ranging from “once or twice a week” to “many times a day” in the past two weeks. Severity, distress, and interference are measured using 11-point numeric rating scales, where 0 indicates “not at all” and 10 indicates “extreme.” Three items assess concomitant cardiac symptoms: 1) pressure, tightness, or pain in the chest; 2) dizziness, faintness, or lightheadedness; and 3) unexplained shortness of breath, with response options of “yes,” “maybe,” or “no.” Participants are also asked if they have previously reported palpitations to a healthcare provider. The total number of endorsed palpitations is summed, as are the responses to the concomitant cardiac symptoms. All other items are evaluated individually.

#### Acceptability feedback questionnaire.

The Acceptability Feedback Questionnaire consists of five items assessing participants’ experiences of wearing the ECG monitor for seven days. Items address comfort while wearing the device, disruption to daily activities, difficulty remembering to press the event button while experiencing palpitations, difficulty with device removal, and willingness to wear the device again. Each item is rated on a 0–10 scale, where 0 indicates an extremely negative experience, and 10 indicates an extremely positive experience. Total scores range from 0 to 50, with higher scores reflecting more positive feedback regarding the device-wearing experience. For any item rated five or below, participants are asked to provide an open-ended explanation describing their discomfort, perceived disruption, difficulty remembering, challenge with device removal, or reluctance to wear the device again.

#### Additional instruments for symptoms, quality of life, and home environment.

While the focus of this study is to examine the feasibility of using an ECG monitor and the relationship between ECG-detected arrhythmias and patient-reported palpitations, we will also collect data on additional symptoms (e.g., fatigue, depressive symptoms, and anxiety) and quality of life for exploratory purposes. These secondary measures will not be the focus of this protocol, but may be examined in future analyses and secondary publications. A summary of these instruments is provided in Table 1.

**Table 1:** Symptoms, Quality of Life, and Home Environment Instability Measures.

Instrument	Description	Scoring	Interpretation
Symptoms			
PROMIS Profile v1.0 Anxiety Short Form 7a [45]	7 items evaluating anxiety symptoms (fearful, anxious, worried, nervous, tense) over past 7 days.	5-point Likert scale (1 = never to 5 = always); raw scores converted to T-scores (M = 50, SD = 10).	Higher T-score = worse anxiety
PROMIS Cognitive Function (8-item [46]	Assesses cognitive function (mental acuity, memory, concentration) over past 7 days.	5-point Likert scale (never to very often); raw scores converted to T-scores (M = 50, SD = 10).	Higher T-score = better cognitive function
PROMIS Profile v1.0 Depression Short Form 8b [47]	8 items assessing depressive symptoms (worthlessness, helplessness, sadness, hopelessness) over past 7 days.	5-point Likert scale; raw scores summed and converted to T-scores.	Higher T-score = greater depression severity
PROMIS Fatigue Short Form 13a (FACIT-Fatigue) [48]	13 items assessing fatigue in the past 7 days.	5-point Likert scale (1 = not at all to 5 = very much); raw scores converted to T-scores.	Higher T-score = worse fatigue
Kessler Psychological Distress Scale (K10) [49,50]	10-item scale assessing frequency of anxiety and depressive symptoms in past 4 weeks.	5-point Likert scale (1 = none to 5 = all the time); scores range 10–50.	Higher score = greater distress; cutoffs: < 20 well, 20–24 mild, 25–29 moderate, ≥ 30 severe
Impact of Event Scale (IES) [51,52]	22 items assessing distress from a traumatic event in past 7 days; subscales: intrusion, avoidance, hyperarousal.	5-point Likert scale (0 = not at all to 4 = extremely); total 0–88.	Higher score = greater trauma-related distress
Insomnia Severity Index [53]	7 items assessing insomnia severity.	5-point Likert scale (0 = none to 4 = very severe); total 0–28.	Higher score = worse insomnia
Perceived Stress Scale (PSS-10) [54]	10 items assessing perceived stress in past month.	5-point Likert scale (1 = never to 4 = very often).	Higher score = greater stress perception
Quality of Life			
Brief Resilience Scale [55]	6 items assessing ability to recover from stress.	5-point Likert scale (1 = strongly disagree to 5 = strongly agree).	Higher score = greater resilience
Quality of Life-Patient Version (QOL-PV) [56]	41 items assessing physical, psychological, social, and spiritual QOL.	0–10 numerical rating scale; mean subscales and total scores calculated.	Higher scores = better QOL
Home Environment Instability			
Confusion, Hubbub, and Order Scale (CHAOS) [57]	15 true/false items assessing disorganization, noise, lack of routine.	Each ‘false’ = 1 point; summed total score.	Higher score = more instability/unpredictability

### Study procedures

**Fig 3:**
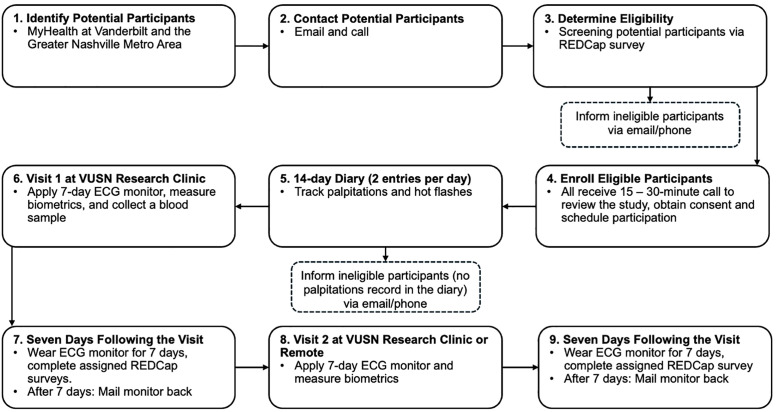
Research procedure flow chart. This figure illustrates the procedures that occur at key points of the study. MyHealth is a participant registry. ECG = electrocardiogram, REDCap = Research Electronic Data Capture System.

Participants will attend two in-person visits approximately one month apart. At each visit, the ECG monitor will be placed over the sternum to capture ECG data (Fig 1). Participants will be instructed to press the ECG button when they feel palpitations. During each 7-day monitoring period, participants will complete the PAT and symptom questionnaires via REDCap. At the end of each monitoring week, participants will complete the Acceptability Feedback Questionnaire regarding their experience wearing the ECG monitor. Afterward, participants will mail back the monitor using the pre-labeled shipping materials provided by Bardy Diagnostics. Fig 3 outlines the steps of the procedure. Detailed visit procedures are described below.

Visit 1:

1. Research staff will measure biometric information, including weight, height, blood pressure, and heart rate.
2. The ECG monitor will be placed on the participant’s chest by research staff.
3. An IRB-approved BardyDx patient instruction sheet containing the following information will be provided:
  a. Participants will receive counseling on permissible activities while wearing the patch, such as showering and exercising, but will be instructed to avoid swimming or immersing the patch during bathing.
  b. Participants will receive guidance on avoiding potential signal disruptions between the sensors and monitors, including electronic blankets, high-voltage areas, and metal detectors.
  c. Participants will be instructed on the “event monitor” button and informed to press it whenever they experience palpitations.
  d. Participants will receive instruction on how to remove the ECG monitor after wearing it for 7 days.
  e. The participant will be instructed on how to return the ECG monitor to the study team using a provided box and shipping tracking code.
4. Blood samples (approximately 5–6 teaspoons) will be collected for future biomarker analysis to investigate the underlying biological mechanisms of palpitations.
5. The second visit will be scheduled for approximately 30 days after participants complete the initial 7-day monitoring period.

Days 1–7 during at-home monitoring:

1. Participants will return home and wear the ECG monitor continuously for 7 days, pressing the event monitor button whenever they experience palpitations. Participants in the comparison group will also wear the ECG monitor continuously for the same duration; however, they will not press the event button unless they experience palpitations during monitoring. The ECG monitor will record heart rate, rhythm, and arrhythmias continuously, regardless of whether participants feel palpitations or press the event button.
2. Participants will be provided with adhesive gel to enhance patch adherence at home. If the patch falls off, participants will be instructed to return to the lab for replacement.
3. Participants will be asked to complete the demographic, PAT, comorbidity, symptoms, and quality of life questionnaires via a secure REDCap form.

Day 8 at the end of at-home monitoring:

4. Upon completion of the 7-day period, participants will remove the ECG monitor and return it to the study team using the provided box and tracking code.
5. Participants will complete an acceptability questionnaire to provide feedback on the experience of wearing the ECG monitor.

Visit 2, Days 1–7 at home monitoring, and day 8 end of monitoring:

1. Approximately one month later, the participant will return to the research site to begin the second 7-day monitoring period.
2. The above procedures will be repeated, excluding questionnaires.

### Primary outcomes

The primary outcome for this study is feasibility, defined as achieving at least 80% recruitment of eligible participants, at least 85% retention through one month, and at least 75% adherence to wearing the ECG monitor for five or more consecutive days.

### Secondary outcomes

Secondary outcomes include the occurrence of palpitations and their association with arrhythmias. Additional characteristics of palpitations will be assessed using the investigator-designed PAT via REDCap to gain a comprehensive understanding of the palpitations experience. Associations between palpitations and other symptoms, such as anxiety, cognitive function, depression, fatigue, psychological distress, stress, insomnia, and perceived stress, as well as quality of life and home environment, will also be evaluated.

### Data management

Data storage and management at the study site will use REDCap, a secure, web-based application for building and managing online surveys and databases [37,38]. After receiving the returned ECG monitors, the PI and research staff will upload the ECG data to the HIPAA-compliant BardyDx server. Summary reports from Bardy will be transferred to each participant’s record in REDCap. Survey data will be collected, managed, and stored in REDCap using study ID numbers. For data cleaning and analyses, de-identified data will be exported from REDCap into Excel or statistical software. To ensure data quality, research staff will regularly monitor data quality through range checks and logic checks, with any discrepancies reviewed and resolved by study personnel. For analysis, de-identified Excel spreadsheets will be stored on a password-protected server. Hard-copy study records will be stored in a locked cabinet within the PI’s office suite, accessible only to the PI and study team, and will be maintained for six years. Blood samples will be labeled with ID numbers and stored in −80°C freezers at the research site building and the university genomics core laboratory. To ensure data quality, research staff will regularly monitor data quality through range checks and logic checks, with any discrepancies reviewed and resolved by study personnel.

### Safety considerations

Participation in this study involves minimal risk. Potential harm is no greater than that encountered during everyday life. Use of the ECG monitors may result in minor skin irritation; however, patches will not be applied to open wounds, lesions, infected, or inflamed skin. Participants will be advised to remove the patch and contact the team if there is redness, severe itching, or allergic symptoms at the patch site. Although extremely rare, improper removal may cause skin damage, which will be mitigated by providing clear removal instructions.

All blood samples will be obtained solely for research purposes using standard blood drawing procedures, performed by trained staff to minimize discomfort. The risks are similar to those of routine blood draws and may include pain, bruising, or infection; however, aseptic precautions will be taken to minimize these risks.

Risks related to data management include potential breaches of confidentiality resulting from online data collection and emotional distress caused by certain questionnaire items. These risks will be clearly explained during informed consent. Participants will be informed that they may skip any questions they find distressing

### Statistical methods

Data analysis will be conducted using IBM SPSS Statistics, STATA, and R, depending on the specific methods being used. The primary outcomes (feasibility, acceptability, and retention) will be evaluated using frequency distributions generated from the study logs and compared to prior benchmarks (80% recruitment of eligible participants, 85% retention through one month, 75% adherence). Descriptive summaries, as appropriate, will be generated of all the secondary outcomes (e.g., prevalence of palpitations, total number and specific types of arrhythmias). We will use generalized linear mixed-effects analyses with link functions appropriate for the nature of each outcome (e.g., log-link for the presence of palpitations, negative binomial for the number of palpitations) to assess the direction and strength of the associations of the number and type of arrhythmias with palpitations. Potential moderating effects (e.g., menopausal status) will be explored to inform targeted recruitment in future work. Given the exploratory nature of this study, emphasis will be placed on effect size and clinical relevance rather than statistical significance. Bootstrapped 95% confidence intervals will be generated around all parameter estimates. Further, comparative analyses using effect statistics will also be conducted to evaluate differences in ECG-detected arrhythmias, laboratory test results (e.g., cardiac biomarkers, such as Troponin I/T, BNP, and NT-proBNP), and variations in genes (e.g., cytokine and potassium channel genes) between participants with and without palpitations.

### Sample size justification

Our proposed recruitment sample size is based on a conservative estimate of the number of participants we can enroll over the 2-year funded study period. We do not have specific a priori effect statistics or hypotheses to justify our sample size based on statistical power; however, the projected sample will suffice to inform feasibility, acceptability, and retention (Aim 1) and to generate stable parameter estimates for future work (Aim 2). Even assuming a conservative attrition rate of up to 20%, we anticipate having analysis samples from at least 60 participants with palpations and 40 without, for our primary comparative analyses. Samples of these size will yield two-sided 95% confidence intervals with margins of error ranging from ± 0.25 to 0.32 SD units and Cohen’s *d* effect statistics as small as 0.51 (80% power, alpha = .05). This Cohen’s d indicates we should be sufficiently powered for moderate or larger effects, which are more likely to be clinically meaningful than smaller effects.

### Ethics statement

The study protocol was approved by the Institutional Review Board at Vanderbilt University (Protocol #231429). All participants will provide written informed consent prior to participation. The study will be conducted in accordance with the ethical standards of the Declaration of Helsinki and institutional guidelines.

## Discussion

This will be the first study to systematically investigate the relationship between self-reported palpitations and ECG-detected arrhythmias in breast cancer survivors. The proposed study will explore palpitations as a clinical indicator of underlying arrhythmia. By utilizing lightweight, wearable ECG monitors, this protocol provides a novel and feasible approach to capturing real-time cardiac rhythm changes in individuals experiencing symptoms.

Self-reported palpitations will be assessed in a more detailed manner than in prior studies. To date, studies assessing palpitations in BCS have relied on a general symptom checklist or menopausal assessment questionnaires [1,2]. In contrast, this study incorporates a purpose-built PAT, designed to more accurately capture the characteristics, severity, and impact of palpitations in this population. This approach may yield more nuanced insights into symptom patterns and their clinical correlates.

We hypothesize that the findings from this study will meaningfully contribute to scientific literature by addressing a critical gap in understanding the association between palpitations and arrhythmia, particularly in BCS. The extended monitoring capacity of the device enhances the precision in capturing the onset and frequency of cardiac arrhythmia in BCS. Identifying when palpitations and/or arrhythmias occur, as well as their frequency, is essential to help BCS and healthcare practitioners compare and implement future treatments. High-frequency episodes may indicate a more severe cardiovascular issue or a higher burden of symptoms, underscoring the value of these data for guiding early detection and management strategies. These data could also be leveraged to train artificial intelligence (AI) models to detect rhythm abnormalities associated with palpitations and characterize the type of rhythm at the time of symptom occurrence. Such advancements could improve the detection of abnormal rhythms and aid in the prevention of severe arrhythmia and adverse cardiovascular outcomes. Because individuals with diagnosed arrhythmias are excluded from the study, we anticipate that underlying arrhythmias will be infrequent. However, identifying even a small proportion of palpitations associated with previously undiagnosed arrhythmias could have important clinical implications, potentially leading to earlier detection, improved patient care, and better outcomes.

This work will also provide important proof-of-concept and feasibility data to support a larger investigation. Findings may inform the development of a clinical risk profile to identify patients at heightened risk for palpitations and arrhythmias during or after cancer treatment. Associations between palpitations and arrhythmias/ECG abnormalities may also establish palpitations as a potential early indicator of underlying cardiovascular problems. These results may also inform appropriate referrals to cardio-oncologists and identify potential therapeutic targets for BCS. Ultimately, this research will support the development of low-cost ECG cardiac assessment strategies and targeted management approaches to enhance symptom recognition, cardiovascular care, and quality of life for BCS.

### Limitations

This study has several limitations. First, although continuous ECG monitoring improves the likelihood of detecting cardiac events, it may not capture all episodes of palpitations or arrhythmias, particularly if participants do not experience symptoms during monitoring periods. The inclusion of a second ECG monitoring session is intended to improve detection. Second, self-reported palpitations may be subject to recall bias or misclassification, which can limit the accuracy of symptom characterization. Third, the use of wearable ECG monitors may introduce variability in data quality due to differences in adherence and device placement. Finally, findings may not be generalizable to all breast cancer populations, especially those with pre-existing cardiovascular conditions.

## Conclusion

Continuous ECG monitoring offers a promising approach to capturing episodes of palpitations and their associations with underlying changes in heart rhythm. This protocol outlines a novel method for studying palpitations and arrhythmias in BCS by combining wearable ECG monitoring with a purpose-built PAT. The inclusion of a control group and the collection of biological samples will provide valuable insights into differences in symptoms, ECG findings, and biological markers. Findings from this study will inform future research focused on early detection, risk stratification, and management of cardiovascular symptoms in this population.
